# Highly Crystalline CVD-grown Multilayer MoSe_2_ Thin Film Transistor for Fast Photodetector

**DOI:** 10.1038/srep15313

**Published:** 2015-10-19

**Authors:** Chulseung Jung, Seung Min Kim, Hyunseong Moon, Gyuchull Han, Junyeon Kwon, Young Ki Hong, Inturu Omkaram, Youngki Yoon, Sunkook Kim, Jozeph Park

**Affiliations:** 1 Multi-Functional Nano/Bio Electronics Lab., Kyung Hee University, Gyeonggi, 446-701, Republic of Korea; 2 Carbon Convergence Materials Research Center, Korea Institute of Science and Technology, Wanju-gun 565-905, Republic of Korea; 3 Department of Electrical and Computer Engineering & Waterloo Institute for Nanotechnology (WIN), University of Waterloo, Waterloo, ON, N2L 3G1, Canada

## Abstract

Hexagonal molybdenum diselenide (MoSe_2_) multilayers were grown by chemical vapor deposition (CVD). A relatively high pressure (>760 Torr) was used during the CVD growth to achieve multilayers by creating multiple nuclei based on the two-dimensional crystal growth model. Our CVD-grown multilayer MoSe_2_ thin-film transistors (TFTs) show p-type-dominant ambipolar behaviors, which are attributed to the formation of Se vacancies generated at the decomposition temperature (650 °C) after the CVD growth for 10 min. Our MoSe_2_ TFT with a reasonably high field-effect mobility (10 cm^2^/V · s) exhibits a high photoresponsivity (93.7 A/W) and a fast photoresponse time (τ_rise_ ~ 0.4 s) under the illumination of light, which demonstrates the practical feasibility of multilayer MoSe_2_ TFTs for photodetector applications.

Smart interface that can provide communication between human beings and digital devices is of great importance in the present and future electronic applications, and intensive research is being carried out in the field of interactive sensing. The latter involves the use of various types of sensors, such as phototransistors that become activated in the presence of light. For instance, active matrix devices consist of at least a driving transistor and switching transistor to afford selective pixel addressing, and active matrix devices incorporating photosensitive thin-film transistors (TFTs) enable the realization of flat panel displays in which specific pixels are locally activated by incident photons^1^. In this regards, smart interface calls for novel active-channel materials to realize high-speed driving transistors and highly sensitive photodetectors.

Layered semiconductors based on transition metal dichalcogenides (TMDs: MX_2_ = Mo, W; X = S, Se, Te) exhibit desirable device characteristics, including high mobility (>100 cm^2^ V^−1^ s^−1^) and large photoresponsivity (~500 A/W)^2^,^3^,^4^,^5^, and mechanical flexibility, which make them attractive for active elements in future interactive electronics. Recently, a significant progress has been made in the synthesis of two-dimensional (2D) semiconductors such as single-layer molybdenum disulfide (MoS_2_) for electronic and optoelectronic applications, which demonstrated high field-effect mobility (>100 cm^2^/V · s) and large photoresponsivity (~500 A/W)^2^,^3^,^4^,^5^, making it attractive for phototransistors. However, the growth of single layers is not favorable for the fabrication of large-area flat panels since it cannot provide sufficient coverage over several square meters under current technologies. In light of this, multilayered structures with high carrier mobility and high photosensitivity are required for the practical large-area sensing applications.

However, indirect-bandgap MoS_2_ multilayers exhibit relatively low photoresponse in TFTs^6^, unlike direct-bandgap MoS_2_ monolayers, although they can provide high field-effect mobility (>100 cm^2^ V^−1^ s^−1^) and small subthreshold swing (~70 mV/decade)^7^,^8^. An advanced local-gate device structure was introduced by Kwon *et al.*^9^ to enhance the photoresponsivity of multilayer MoS_2_ phototransistors. On the other hand, it is known that MoSe_2_ can provide higher photoresponsivity compared to MoS_2_ due to the quantum confinement effect during the bandgap transition^10^, which implies that using an advanced device structure may not be needed for MoSe_2_ phototransistors to achieve high sensitivity. In addition, Choi *et al.*^6^ reported that TMD multilayers have an advantage over the monolayers that photoresponse is achievable over a broad range of the electromagnetic spectrum from ultraviolet to near infrared. Therefore, multilayer MoSe_2_ can be a strong contender for an active channel material of future phototransistors.

In this study, we present the chemical vapor deposition (CVD) growth of MoSe_2_ multilayers at a relatively high pressure. The CVD methods reported up to date involve low-pressure deposition with slow nucleation rates^11^,^12^,^13^,^14^,^15^, resulting in triangular single layers terminated by either transition metal (*e.g.*, Mo) or chalcogen atom (*e.g.*, Se) for TMDs. In contrast, we demonstrate that indirect-bandgap MoSe_2_ multilayers can be grown by using a high-pressure CVD method. Based on the two-dimensional nucleation theory, a relatively high pressure at a fixed temperature can induce a large nucleation rate before film growth occurs, and thus, the formation of multilayers is promoted. The microstructure of hexagonal MoSe_2_ grains is examined using X-ray diffraction (XRD), high-resolution transmission electron microscopy (TEM) and Raman spectroscopy. Our multilayer MoSe_2_ TFTs exhibit ambipolar behaviors with high photoresponsivity (93.7 A/W) and reasonably large field-effect mobility (~10 cm^2^/V · s). This highly crystalline and photo-responsive multilayer MoSe_2_ is anticipated to be used in a myriad of potential applications for interactive electronics.

## Results and Discussion

**Figure 1:**
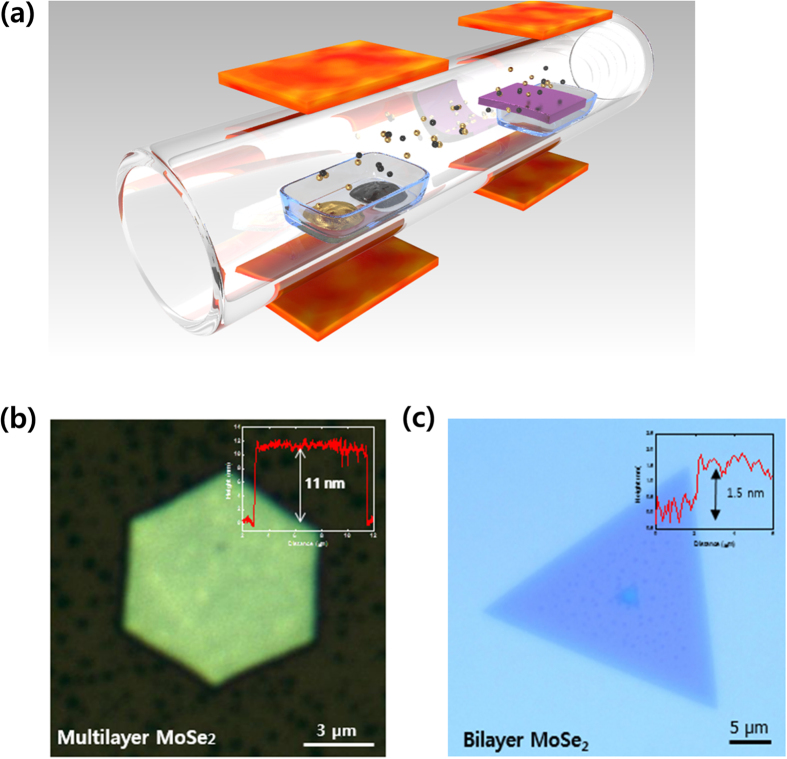
Synthesis of the hexagonal multilayer MoSe_2_ nanoparticles. (**a**) Schematic process for the synthesis of the hexagonal multilayer MoSe_2_ nanoparticles with MoO_3_ and Se sources. (**b**) Optical microscope images of the as-grown MoSe_2_ multilayers and (**c**) as-grown MoSe_2_ bilayer on SiO_2_/Si. The insets show the AFM height profile of the as-grown MoSe_2_ multilayer and bilayer to measure the thickness.

Figure 1(a) shows a schematic CVD process for the growth of hexagonal multilayer MoSe_2_. Unlike recent studies^16^,^17^, MoO_3_ and Se are contained in the same alumina boat located at the upper stream of the CVD furnace. A SiO_2_/Si substrate is placed downstream, facing up on the alumina boat and is left to react with the source materials. The furnace temperature was ramped up to 800 °C and 750 °C for the sources and the substrate, respectively, at a rate of 15 °C/min in an evacuated ambient. The vaporized source molecules are efficiently transferred to the substrate surface by the temperature gradient with the aid of carrier gases such as Ar and H_2_. During the chemical reaction between MoO_3_ and Se, H_2_ gas acts as a catalyst^18^. After the CVD process, hexagonal MoSe_2_ crystallites of various thickness and size were obtained. Figure 1(b) shows an optical image of an as-grown hexagonal MoSe_2_ grain with a thickness of approximately 11 nm, measured by atomic force microscopy (AFM). In comparison, Fig. 1(c) shows the height profile of an as-grown MoSe_2_ bilayer using a relatively low pressure (<760 torr). While the thickness of a MoSe_2_ monolayer lies generally between 0.6 nm and 1 nm^18^,^19^,^20^, hexagonal MoSe_2_ multilayers obtained in the present work show an average thickness of approximately 12 nm with the size in the range of 4–9 μm. The shape of film depends on the film thickness, which is attributed to the difference in the edge formation energy between Mo-edge or Se-edge termination. In single or few layered MoSe_2_ films, the difference in the edge formation energy depending on Mo-edge (100) or Se-edge 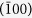 termination results in triangular shaped grains, but since the effect of specific edge termination is cancelled out by alternating Mo- and S-edges in multilayer MoSe_2_ films, forming a hexagonal shape is expected from the hexagonal crystal structure of MoSe_2_ film^21^.

The relatively large flow rates of injected Ar and H_2_ gases in this work are anticipated to increase the pressure in the chamber. Based on two-dimensional nucleation theory^22^, a relatively high pressure induces large nucleation rates, and thus multiple nuclei may form on already existing ones, resulting in multilayered structures. The rate of forming stable two dimensional nuclei is usually described by the following equation:


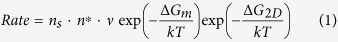


where *n*_s_ is the concentration of single adsorbed atoms on a closed packed surface, n* is the concentration of atoms in contact with a two-dimensional nucleus with a critical size, *ν* the vibration frequency, Δ*G*_m_ the free energy of activation for a surface diffusion jump, and Δ*G*_2D_ the free energy of formation of a critical nucleus. The latter is again represented by the following relationship:


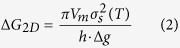


where *V*_*m*_ is the molar volume of the solid phase, *σ*_*s*_(*T*) is the free energy of step formation per unit length, *h* the height of the monolayer step, and Δ*g* the difference of molar free energy between the vapor and solid phases in the case of CVD deposition. The growth of MoSe_2_ layers is attributed to the condensation of vaporized MoO_3_ and Se radicals on the substrate, forming Mo_x_Se_2-x_ nuclei. As the pressure increases, the *n*_s_ also increases, resulting in the enhancement of the nucleation. Another effect of higher pressure is the increase of Δg, as more MoSe_2_ molecules assemble on the substrate surface. A relatively large Δg value results in decreased Δ*G*_2D_ barrier, thereby contributing to higher nucleation rate rather than growing from the existing nuclei. It is thus expected that the growth of multilayers is attributed to the formation of additional nuclei to the existing ones with a large nucleation rate. In order to confirm this theory, relatively thin few-layered MoSe_2_ films were grown at a lower pressure. Figure 1(c) is an optical image of a 1.5 nm-thick MoSe_2_ grain, which was synthesized at a reduced pressure using lower gas flow rates (Ar: 50 sccm, H_2_: 10 sccm). The pressure-dependent film thickness was also demonstrated previously for graphene and graphite layers: Graphite was obtained at relatively high pressures^23^, while lower pressures were used for the formation of graphene^24^.

**Figure 2:**
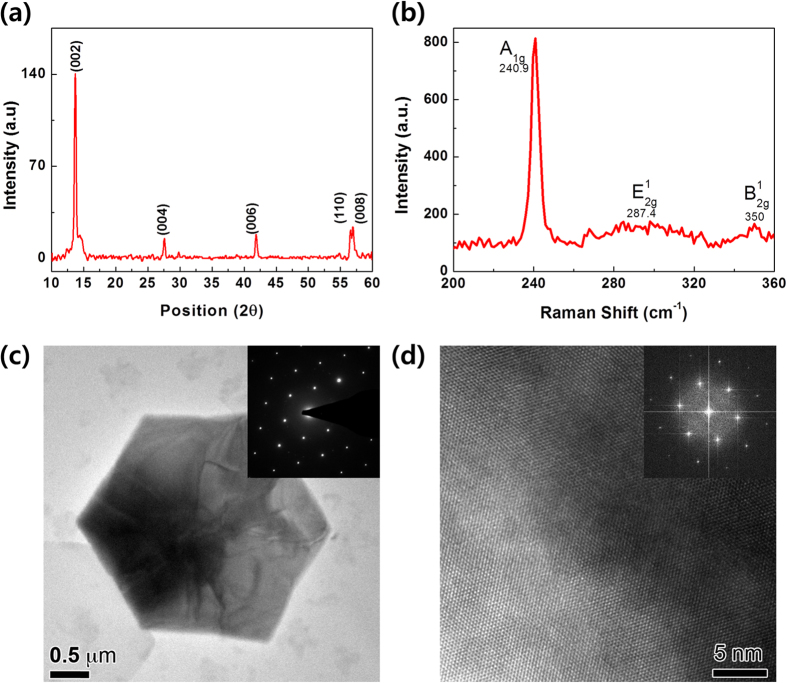
Spectroscopic analyses of CVD-synthesized hexagonal MoSe_2_. (**a**) XRD pattern of MoSe_2_ layers indicating the preferential growth in the (002) direction with intense (002), (004), (006), and (008) peaks, along with the presence of a weak (110) peak. (**b**) Raman spectra of MoSe_2_ with in-plane (E^1^_2g_) and out-of-plane (A_1g_) vibration modes. (**c**) Plan-view low magnification and (**d**) high resolution TEM images of an as-synthesized MoSe_2_ flake. The inset in (**c**) is a SAED pattern form within the flake and the inset in (**d**) is a FFT pattern from the entire area of (**d**).

Figure 2(a) shows the XRD pattern of our MoSe_2_ having a clear hexagonal monocrystalline structure^25^. The symmetry of 2H MoSe_2_ belongs to the space group D^4^_64_ (P6_3_/mmc), which reveals characteristic peaks at 2θ = 13.72°, 27.62°, 41.88°, 56.59° and 56.97° corresponding to the (002), (004), (006), (110) and (008) diffractions for MoSe_2,_ respectively. The most intense (002) peak indicates the preferential growth of the MoSe_2_ crystallites in the (002) direction. To assess the presence and quality of the MoSe_2_ films, Raman spectroscopy with a laser wavelength of 514.5 nm was carried out. The as-grown MoSe_2_ atomic layers in this study exhibit several signatures of MoSe_2_ in the Raman shift ranging from 200 cm^−1^ to 360 cm^−1^. Along with the out-of-plane A_1g_ mode (Fig. 2(b)) the atomic vibrations corresponding to several less prominent modes including the E_2g_^1^ (in-plane) and B_2g_^1^ modes are in agreement with information provided in former reports available in the literature^10^,^26^,^27^. The latter modes have significantly lower intensities compared to the most intense out-of-plane A_1g_ peak located at 240.9 cm^−1^. The typical Raman active modes, *i.e.* the broad & weak E_2g_^1^ peak located at 287.4 cm^−1^ and the B_2g_^1^ peak located at 350 cm^−1^, are observed. The B_2g_^1^ mode is a shear mode corresponding to the vibration of two rigid layers against each other and appears at relatively low frequencies. The A_1g_ mode is an out-of-plane vibration involving only the chalcogen atoms (Se) while the E_2g_^1^ mode involves the in-plane displacement of the transition metal (Mo) and chalcogen atoms (Se). Figure 2(c) shows a plan view low magnification TEM image of an as-synthesized MoSe_2_ flake about 3–4 μm large. The inset in Fig. 2(c) represents a selected area electron diffraction (SAED) pattern taken from within the flake. The pattern reflects well the hexagonal monocrystalline structure along the (002) zone, confirming the XRD results. Figure 2(d) consists of a high resolution TEM image of the thin edge of a MoSe_2_ flake, and the inset is a fast Fourier transform (FFT) pattern from the entire area of the figure. The above analyses clearly indicate the presence of a highly crystalline hexagonal MoSe_2_ phase.

**Figure 3:**
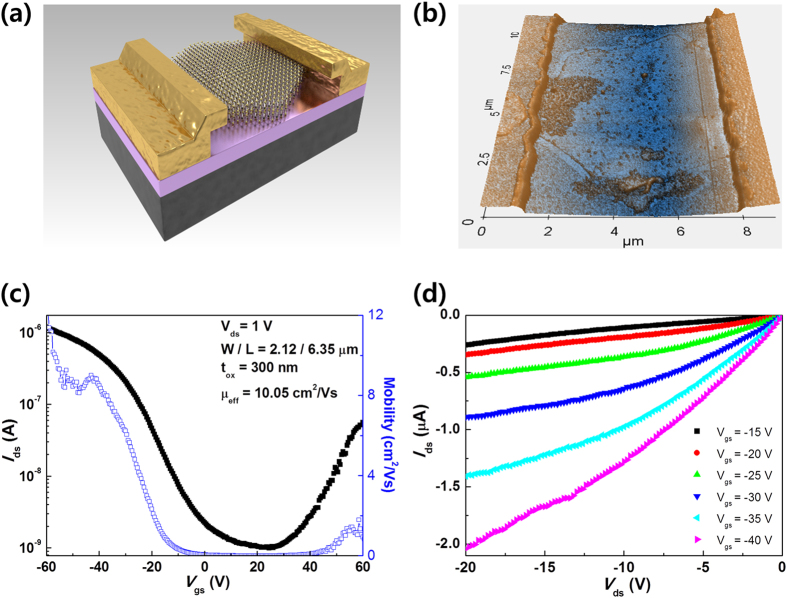
Thin-film transistor based on the CVD-synthesized hexagonal MoSe_2_ multilayer. (**a**) 3D schematic structure for TFT based on hexagonal multilayer MoSe_2_ film. (**b**) 3D topography AFM image of the hexagonal MoSe_2_ TFT with a channel length of 6.35 *μ*m. (**c**) Transfer (*I*_ds_ − *V*_gs_) curve and field-effect mobility (*μ*_eff_) of the hexagonal MoSe_2_ TFT (−60 ≤ *V*_gs_ ≤ 60 V at *V*_ds_ = 1 V). (**d**) Output characteristics of the respective device (−20 ≤ *V*_ds_ ≤ 0 V, −15 ≤ *V*_gs_ ≤ −40 V in steps of −5 V).

Figure 3(a) shows a three-dimensional (3D) schematic of a MoSe_2_ TFT device. The electrodes consist of Ti as an adhesion layer and Au, and the devices were annealed at 200 °C under atmospheric conditions with Ar and H_2_ in order to reduce contact resistance and remove the photoresist remnants. Figure 3(b) shows a 3D topography AFM image, where the thickness of the MoSe_2_ channel is approximately 20 nm. Figure 3(c) shows the typical drain current (*I*_ds_) versus gate voltage (*V*_gs_) characteristics of the TFT and the extracted mobility values are also plotted as a function of *V*_gs_. The field effect mobility (*μ*_eff_) was calculated using the following relationship; *μ*_eff_ = g_m_ * *L*/(*WC*_ox_*V*_ds_), where *L* is the channel length (~6.35 μm), *W* is the channel width (~2.12 μm), *C*_ox_ is the capacitance of the gate insulator per unit area, and *V*_ds_ the applied drain-source voltage (1 V). The maximum transconductance, *g*_*m*_, was extracted to be approximately 54 μS. The devices exhibit an ambipolar behavior with a predominant p-type characteristic, with the highest *μ*_eff_ being approximately 10 cm^2^/V · s and an ON/OFF current ratio of ~10^3^, while the electron mobility was extracted as 2.14 cm^2^/V · s, at the positive region. The output characteristics (*I*_ds_ − *V*_ds_) were measured in the negative *V*_ds_ range (Fig. 3(d)), which also depict clear p-type behaviors.

**Figure 4:**
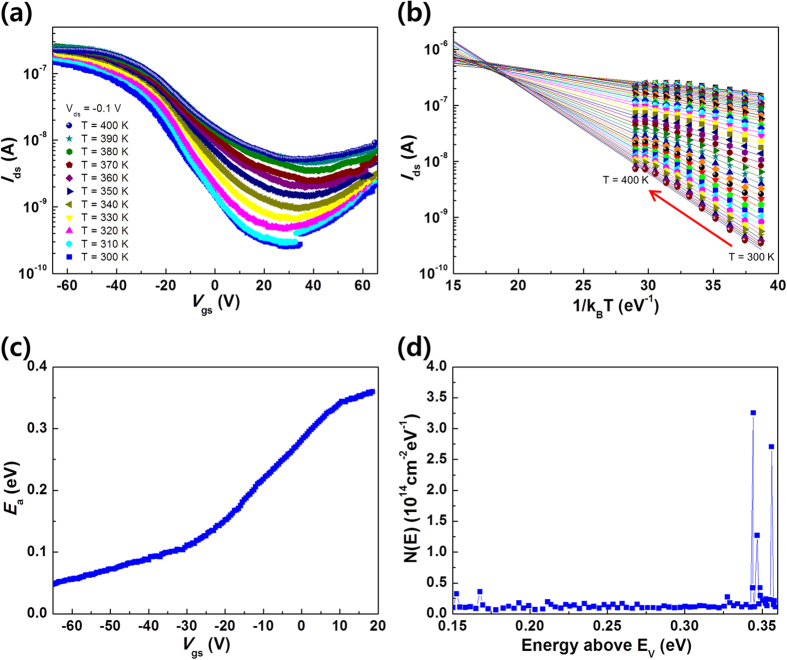
Temperature-dependent behavior and density-of-state measurement according to the Meyer-Neldel rule. (**a**) Transfer characteristics with temperatures from 300 to 400 K in steps of 10 K at *V*_ds_ = 0.1 V. (**b**) Temperature dependence of the drain current (*I*_ds_) as a function of 1/*k*_*B*_*T*. (**c**) Activation energy (*E*_a_) extracted using the Meyer-Neldel rule as a function of gate voltage. (**d**) Density of sub-gap states calculated for the p-type MoSe_2_ TFT as a function of energy above the valence band (*E*_*V*_). A large density can be observed ~0.35 eV above *E*_*V*_.

It is worth investigating the characteristics of p-type MoSe_2_ TFTs, since most recent studies on MoSe_2_ TFTs exhibited n-type behaviors^25^,^28^. Here, it is hypothesized that the band structure of hexagonal MoSe_2_ multilayers is influenced by a large density of trap sites created by an annealing process during the CVD growth. A recent study demonstrated that irradiation with MeV α particles or thermal annealing at sub-decomposition temperature (~600 °C) creates anion vacancies in TMD materials such as MoS_2_, MoSe_2_, and WSe_2_ 29. Similarly, we can expect that Se vacancies may be created in the present MoSe_2_ film when the decomposition temperature (~650 °C) is reached after the CVD growth for 10 min. Such defects may also significantly affect the electrical behavior through Fermi level pinning, making the theoretical prediction of the Schottky barrier height (Φ_*Bn*_ = Φ_*m*_ − *χ*, where Φ_*m*_ and *χ* are the metal work function and the semiconductor’s electron affinity, respectively) ineffective^30^,^31^. In order to extract the sub-gap states, a temperature-dependent analysis was performed^32^,^33^. Figure 4(a) shows the *I*_*ds*_ − *V*_*gs*_ characteristics at different temperatures between *T* = 300 and 400 K. Figure 4(b) depicts the thermally activated current at different *V*_*gs*_ values, as a function of 1/*k*_*B*_*T* (where *k*_*B*_ is the Boltzmann constant). The Arrhenius equation is used to describe the current response to the temperature as 
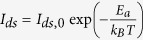
, where *I*_*ds*,0_ is a prefactor and *E*_*a*_ is the activation energy^32^. The variation of *E*_*a*_ at different gate voltages is shown Fig. 4(c), from which the density of sub-gap states can be obtained by 
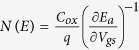
 where *q* is the elementary charge. Figure 4(d) shows a large density of sub-gap states in the band gap near *E*_*V*_ + 0.35 eV (*E*_*V*_ being the valence band maximum), which is 0.07–0.2 eV below the midgap energy (*E*_*m*_), since the band gap of bulk MoSe_2_ is reported to be 0.84–1.1 eV^10^,^34^,^35^. Therefore, at the Ti/Au metal–MoSe_2_ junction, Fermi level pinning^36^ caused by the gap states is expected to occur in such a way that the Schottky barrier height for holes becomes smaller (Φ_*Bp*_ ≈ *E*_*g*_/2 − 0.14 eV) than that for electrons (Φ_*Bn*_ ≈ *E*_*g*_/2 + 0.14 eV), resulting in p-type-dominant ambipolar behavior as shown in Fig. 3(c).

**Figure 5:**
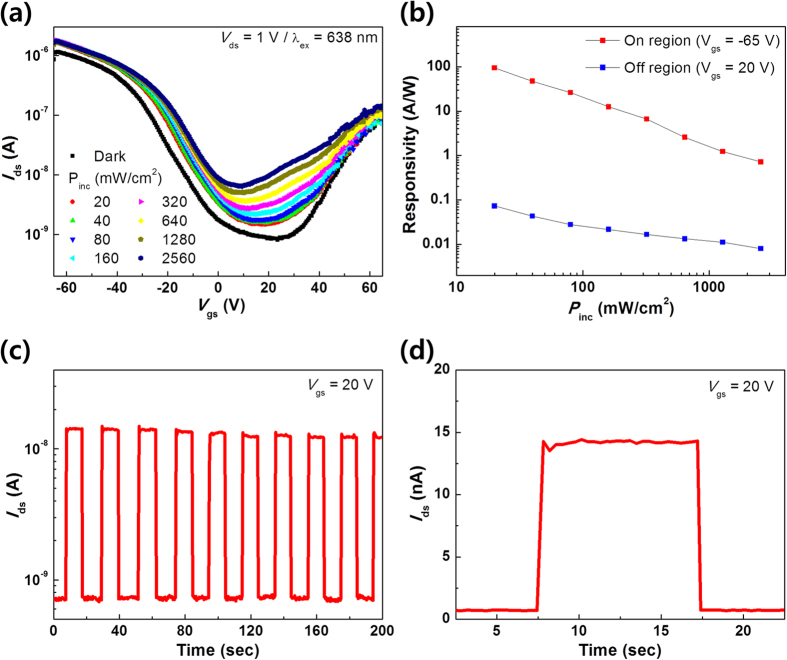
Photoresponsive behavior of hexagonal MoSe_2_ TFT. (**a**) Comparison of transfer characteristics (*I*_ds_ − *V*_gs_) in the dark and under illumination with different optical power densities (*λ*_ex_ = 638 nm, *P*_inc_ = 20, 40, 80, 160, 320, 640, 1280 and 2560 mW/cm^2^). (**b**) Responsivity of the device in logarithmic scale in the on (*V*_gs_ = −65 V) and off (*V*_gs_ = 20 V) regions. (**c**) Switching behavior (*I*_ds_ − Time) of the photodetector at *V*_gs_ = 20 V. The device was switched on and off with the laser (*λ*_ex_ = 638 nm) at an interval of 10 s (a period of 20 s). (**d**) One cycle of the laser pulse, showing the drain current in the off region at *V*_gs_ = 20 V on a linear-scale of (**c**).

In order to investigate the optoelectronic properties of MoSe_2_ multilayers, the photoresponse of the TFTs were examined. Figure 5(a) shows the transfer characteristics under illumination with a 638-nm laser as a function of *V*_*gs*_ at various incident power densities (from 20 to 2560 mW/cm^2^). The drain bias (*V*_ds_) was fixed at 1 V. The photoresponsivity is defined as *R* = *I*_ph_/(*P*_inc_*S*), where *R* is the photoresponsivity, *I*_ph_ (=*I*_total_ − *I*_dark_) is the photo-induced photocurrent, *S* is the channel area of the device and *P*_inc_ (W/cm^2^) = *P*_tot_/*A*_laser_ (*A*_laser_ is the area of laser spot) is the incident power density. The calculated photoresponsivity values with respect to the incident power density are shown in Fig. 5(b). The maximum photoresponsivity of 93.7 A/W was achieved at *V*_gs_ = −65 V with the lowest incident power density (20 mW/cm^2^). Notably, this is the highest value reported up to date concerning CVD-grown MoSe_2_ TFTs, and the photoresponsivity is also comparable to that of mechanically exfoliated MoSe_2_ TFT^37^,^38^,^39^,^40^. Recently, Tongay *et al.*^29^ reported that the trap sites related to the anion vacancies, whose energy levels are located between the conduction band (CB) and the valence band (VB), can enhance the photoresponsive properties of TMD. Our temperature-dependent measurements (Fig. 4) also exhibit a large density of sub-gap states in as-grown multilayer MoSe_2_, which can be the origin of the large photoresponsivity in our multilayer MoSe_2_ TFTs, by providing excess photo-induced hole carriers upon exposure to light. To evaluate the devices for potential application as photodetectors, the photoswitching behavior was also examined. Figure 5(c) shows the time-resolved drain current when the incident laser is switched on and off with a power density of 2560 mW/cm^2^ and a time period of 20 s, while the gate voltage is fixed at 20 V. As shown in Fig. 5(d), the photoswitching behavior consists of a relatively short rising time (τ_rise_ ~ 0.4 s) and a short decay time (τ_decay_ ~ 0.2 s) that form a nearly-ideal rectangular pulse. Such characteristics indicate that MoSe_2_ devices are promising for photodetector applications.

In conclusion, we presented highly sensitive phototransistors based on CVD-grown hexagonal MoSe_2_ multilayers. A relatively high pressure (>760 Torr) in the CVD chamber, originating from a large flow rate of injected Ar and H_2_ gases, stimulates the formation of multiple nuclei, resulting in multilayered MoSe_2_ nanosheets. The decomposition temperature of MoSe_2_ (~650 °C) was reached after the CVD growth for 10 min, which is supposed to induce Se vacancies in MoSe_2_. Such defects are believed to be the origin of the observed ambipolar conduction in our multilayer MoSe_2_ TFTs, through the Fermi level pinning at the metal-MoSe_2_ semiconductor interface. Moreover, the Se vacancies can enhance the optical properties of MoSe_2_ devices, which were manifested by the highest photoresponsivity (93.7 A/W) reported to date and a fast response time (τ_rise_ ~ 0.4 s) to the incident light. The results presented in this work will open up a new route for the fabrication of interactive electronics incorporating active-matrix displays and photosensing devices.

## Methods

### Synthesis of hexagonal MoSe_2_ particles

MoSe_2_ films were synthesized inside a CVD furnace composed of two zones; a 2-inch diameter horizontal tube furnace with a 2-inch diameter quartz tube. MoO_3_ powders (Sigma Aldrich 99.5% purity) were placed on the right side of an alumina boat. Se powders (Sigma Aldrich 99.5% purity) were placed on the left side of the same boat. A 3 × 3 cm Si wafer with 300 nm SiO_2_ grown on it was cleaned using acetone and isopropyl alcohol. The substrate was placed on top of the boat that is facing up. This configuration positions the sources in the hot zone and the substrate in the cold zone. The hot and cold zones were heated up to 800 °C and 750 °C, respectively, at 15 °C/min. The reaction chamber was constantly filled with 120 sccm Ar and 20 sccm H_2_ gas for 20 min to allow the reaction to take place. After MoSe_2_ growth for 20 min, the chamber was slowly cooled down to 650 °C for 10 min to generate Se vacancies and quenched to room temperature.

### Synthesis of triangular bilayer MoSe_2_ particles

The substrate and sources were put on the same place of the multilayer growth method. The critical differences between the two growth methods were injection gas quantity and the chamber pressure. The chamber was created a vacuum state using a pump. After purging, the gases, 50 sccm Ar and 10 sccm H_2_, were constantly injected in the reaction chamber during the growth process. The reaction chamber pressure was kept around the atmospheric pressure. The other processes were same as the multilayer growth method.

### Device fabrication

For the source and drain electrode, Ti (20 nm) was first deposited as an adhesion layer and Au was then grown (300 nm) using e-beam evaporation at room temperature. The electrodes were patterned by photolithography, resulting in a channel length of 6.35 μm. The as-fabricated device was annealed at 200 °C under atmospheric conditions for 2 h while being exposed to 100 sccm Ar and 10 sccm H_2_ gas to eliminate the photoresist residue and to reduce the contact resistance.

### Characterization

Optical images of the hexagonal MoSe_2_ TFT were taken using an optical microscope (BX51M, Olympus Co., JAPAN) with white light (100 W halogen lamp, U-LH100-3) in bright field imaging mode and a 50× objective lens. The TEM images and diffraction patterns were obtained using a transmission electron microscope (FEI Tecnai^TM^ F20) operated at an acceleration voltage of 200 kV. For TEM sample preparation, the sample was cut to a 3 mm disk and the backside of the sample was hand-polished and dimpled down to about 5–10 μm at the center of the sample. Then, the sample was ion-milled from the backsides at a 4.5° angle and at 4.5 kV using a Gatan PIPS^TM^ until the small hole at the center of the sample was made. The topography of the MoSe_2_ phototransistor was measured using an AFM (XE7 Atomic Force Microscope, Park Systems, South Korea) under non-contact mode with a 0.2 Hz scan rate. The electrical characteristics of the phototransistor were measured using a parameter analyzer (Keithley 4200 SCS) at room temperature. The photoresponsive properties of the MoSe_2_ phtotransistor were evaluated using an illumination system composed of a Nikon Ti-e microscope with an Acton SP2300 spectroscope and a Zolix TLS3900x-500 tunable light source.

## Additional Information

**How to cite this article**: Jung, C. *et al.* Highly Crystalline CVD-grown Multilayer MoSe_2_ Thin Film Transistor for Fast Photodetector. *Sci. Rep.* **5**, 15313; doi: 10.1038/srep15313 (2015).
